# Knee rotation significantly increases measured tibial tubercle‐trochlear groove distance in female patients with anterior knee pain: Findings after rotational measurement correction

**DOI:** 10.1002/jeo2.70681

**Published:** 2026-03-09

**Authors:** Vicente Sanchis‐Alfonso, Jesús Castellano‐Curado, Marco Gulmini, Cristina Ramirez‐Fuentes, Robert A. Teitge, Julio Doménech‐Fernández

**Affiliations:** ^1^ Department of Orthopaedic Surgery Hospital Arnau de Vilanova Valencia Spain; ^2^ Department of Orthopaedic Surgery Hospital Reina Sofía Cordoba Spain; ^3^ Department of Radiology Hospital Universitario y Politécnico La Fe Valencia Spain; ^4^ Department of Orthopaedic Surgery Wayne State University Detroit MI USA

**Keywords:** anterior knee pain, femorotibial rotation, knee rotation angle, patellofemoral pain, TT‐TG distance

## Abstract

**Purpose:**

To evaluate the influence of knee rotation angle (KRA) on tibial tubercle‐trochlear groove (TT‐TG) distance measured by computed tomography (CT) in female anterior knee pain (AKP) patients. To assess how correcting for knee rotation affects TT‐TG values. To correlate rotational corrected TT‐TG with the tibial tubercle‐posterior cruciate ligament (TT‐PCL) distance and tibial tubercle lateralisation (TTL) ratio.

**Methods:**

A retrospective study was performed on 100 consecutive female AKP patients (183 lower limbs) who underwent torsional CT scans. TT‐TG distance was measured and KRA was determined. Rotational‐corrected TT‐TG (RC TT‐TG) values were calculated by neutralising knee rotation. Moreover, TT‐PCL distance and TTL were measured. Two orthopaedic surgeons independently performed all measurements. Spearman correlation and linear regression analyses were used to evaluate the relationship between KRA and TT‐TG changes.

**Results:**

The mean native TT‐TG was 17.22 ± 4.20 mm, mean KRA was 8.81 ± 5.08° and mean corrected TT‐TG was 9.34 ± 4.70 mm. The difference between noncorrected and corrected TT‐TG distance showed a very strong positive correlation with KRA (*ρ* = 0.934, *p* < 0.001). Linear regression analysis demonstrated that KRA explained 73.3% of the variability in TT‐TG changes (*R*² = 0.733), with TT‐TG decreasing by approximately 0.84 mm for each degree of knee rotation. TT‐TG distance showed a moderate positive correlation with TT‐PCL (*ρ* = 0.515, *p* < 0.001). In contrast, rotational‐corrected (RC) TT‐TG demonstrated a stronger correlation with TT‐PCL (*ρ* = 0.644, *p* < 0.001). The Spearman correlations show a moderate positive association between the normal TT‐TG and TTL (*ρ* = 0.451, *p* < 0.001), while the RC TT‐TG exhibits a stronger correlation with TTL (*ρ* = 0.539, *p* < 0.001).

**Conclusion:**

Knee rotation is a significant factor affecting TT‐TG measurement. Measuring TT‐TG after correcting knee rotation to neutral provides a more accurate estimation of this measurement. RC TT‐TG correlates more closely with rotation‐independent tibial‐based measurements.

**Level of Evidence:**

Level IV.

## Graphical abstract

TT‐TG distance increases with knee rotation, while rotational correction reduces it by ~ 0.84 mm per degree, markedly decreasing pathological values.

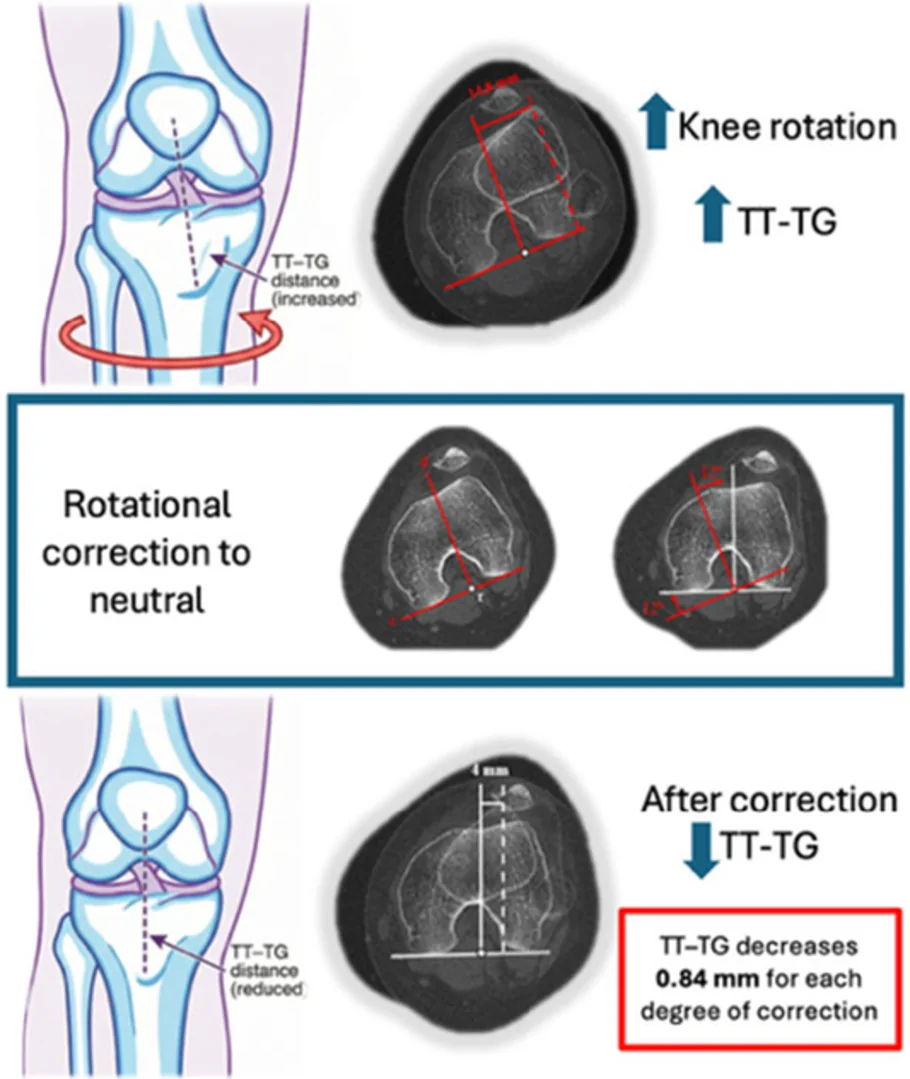

**Abbreviations**
AKP: anterior knee pain
CT: computed tomography
ICC: intraclass correlation coefficient
KRA: knee rotation angle
PACS: picture archiving and communication system
RC TT‐TG: Rotational‐corrected tibial tuberosity‐trochlea groove
TT: tibial tubercle
TT‐TG: tibial tuberosity‐trochlea groove
ΔTTTG: difference in TT‐TG

## INTRODUCTION

Currently, the tibial tubercle‐trochlear groove (TT‐TG) distance is one of the most widely used radiological parameters for determining the need for surgery in patellofemoral disease [6]. Most authors agree that values greater than 20 mm, measured by computed tomography (CT), are pathological and indicate the tibial tubercle (TT) is lateralized from its anatomically normal position. Such elevated values are a classic criterion for recommending TT osteotomies in patients with patellofemoral disease [17].

However, several studies have questioned the validity of using TT‐TG distance alone, as this measurement is influenced by multiple factors, including structural factors (e.g., femoral anteversion, tibial torsion, trochlear dysplasia and patellar height), functional factors (e.g., femorotibial rotation and patellar tilt) and positional factors (e.g., knee flexion during imaging) [1, 3, 4, 7, 11, 12, 18, 22, 24]. Of particular interest is the growing interest in the role of knee rotation angle (KRA) as a determinant of TT‐TG distance. It has been shown that TT‐TG distance can vary by approximately 0.5 mm for each degree of knee rotation, demonstrating that small angular changes can significantly influence the clinical interpretation of this measurement [16]. Similarly, cadaver studies and imaging studies in patients with patellofemoral instability have demonstrated a relationship between knee rotation and TT‐TG distance [2, 13, 21]. This suggests that the apparent increase in TT‐TG observed in some patients may be due to increased knee rotation rather than true anatomical lateralisation of the TT. To provide a tibial‐based measurement independent of femorotibial rotation, the tibial tubercle‐posterior cruciate ligament (TT‐PCL) distance and the tibial tubercle lateralisation (TTL) ratio were introduced [14, 20].

It has been hypothesised that the TT‐TG distance is significantly influenced by knee rotation. External tibial rotation of the tibia on the femur will move the TT laterally, so the measured TT‐TG distance is increased. In some cases, this could lead to an overestimation of the distance, and therefore, TT‐TG distance should not be used as an indication for surgery. Our second hypothesis is that rotational‐corrected TT‐TG distance should be correlated more closely with rotation‐independent tibial‐based measurements than normal TT‐TG distance. The first objective of this study is to evaluate the influence of the KRA on the TT‐TG distance measured by CT in patients with anterior knee pain (AKP), as well as to determine how the correction of this rotation affects the final value of the TT‐TG distance. The second objective of this paper would be to correlate rotational‐corrected TT‐TG with the TT‐PCL and TTL.

## MATERIALS AND METHODS

### Participants and study design

This study was approved by our institutional review board (CEIm Hospital Arnau de Vilanova, Valencia, Spain # PI 21_2024). A retrospective study including 100 consecutive female patients (183 lower limbs) evaluated for AKP resistant to an adequate conservative treatment was performed. All participants were caucasian females. For AKP patients with disabling pain and severe disability not responding to conservative treatment, weight‐bearing whole‐limb anteroposterior view radiograph, CT for evaluation of torsional abnormalities and magnetic resonance imaging (MRI) were performed in all of them as part of a routine radiographic workup. In all cases, the torsional CT study was performed for strictly clinical reasons following the diagnostic protocol of the Knee Unit of our Department. In our cohort, females predominated, and therefore, we include females only to reduce potential bias in the results. Patients with a history of previous surgery on the affected knee, periarticular fractures or congenital abnormalities that could alter the anatomy relevant to the measurements were excluded from the study.

### CT protocol

CT images were acquired using a high‐spatial‐resolution 256‐detector row CT scanner (Brilliance iCT; Philips). Patients were positioned supine with the hip and knee fully extended and their feet in 15° of external rotation. Three separate CT scans were obtained for each patient, a hip scan—from the upper edge of the femoral heads to immediately distal to the lesser trochanters, a knee scan—from the upper edge of the patella to immediately distal to the TT, and an ankle scan—including the tibial plafond and both malleoli. The raw data sets acquired were 64 × 0.625 mm collimation, 0.5 s rotation time, 0.9 mm slice reconstruction thickness, 0.45 pitch, 120 kV and automated mAs control. Torsional measurements were performed manually using the imaging tools on the advantage workstation 4,5 software (GE HealthCare) integrated in the picture archiving and communication system. All images in the present study have been previously anonymised. Once anonymisation was carried out, two orthopaedic surgeons (J.C.‐C. and M.G.) performed all measurements independently using calibrated digital measurement software and included them in the database.

### CT measurements

**Figure 1:**
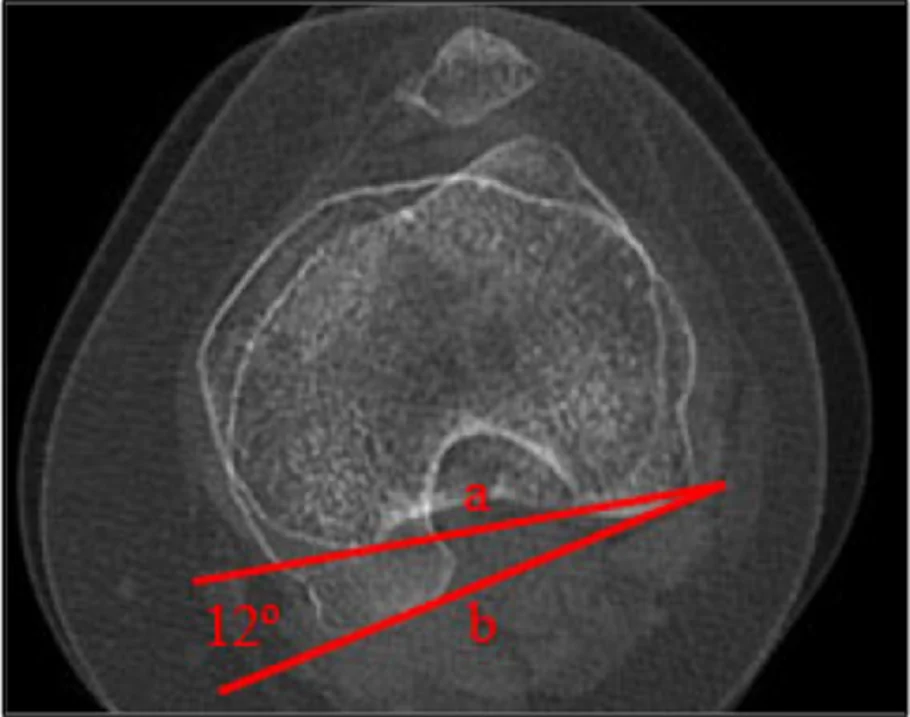
Knee rotation angle (KRA) determined using the Takai method [19]. Line (a) correspond to the tibial bicondylar line. Line (b) correspond to the posterior femoral condylar line. KRA (12°) is the angle measured between the lines (a) and (b).

**Figure 2:**
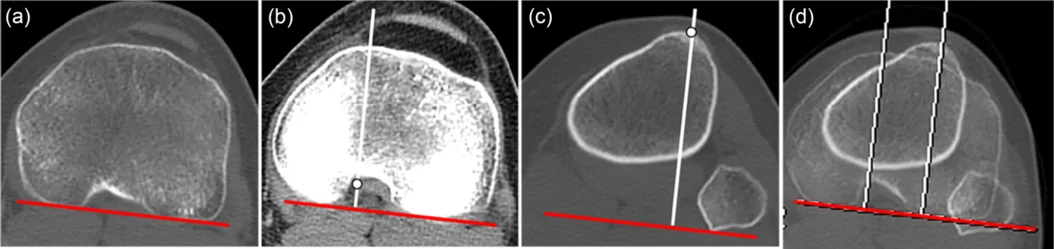
TT‐PCL distance measurement according to the technique described by Seitlinger et al. (a) Shows the dorsal condylar line of the tibia (red line). (b) Shows the line that passes through the medial border of the posterior cruciate ligament (white line) and is perpendicular to the dorsal condylar line. (c) Shows the line that passes through the centre of the insertion of the patellar tendon on the tibial tubercle (white line) and is perpendicular to the dorsal condylar line. (d) The distance between both white lines is the TT‐PCL distance. TT‐PCL, tibial tubercle‐posterior cruciate ligament

**Figure 3:**
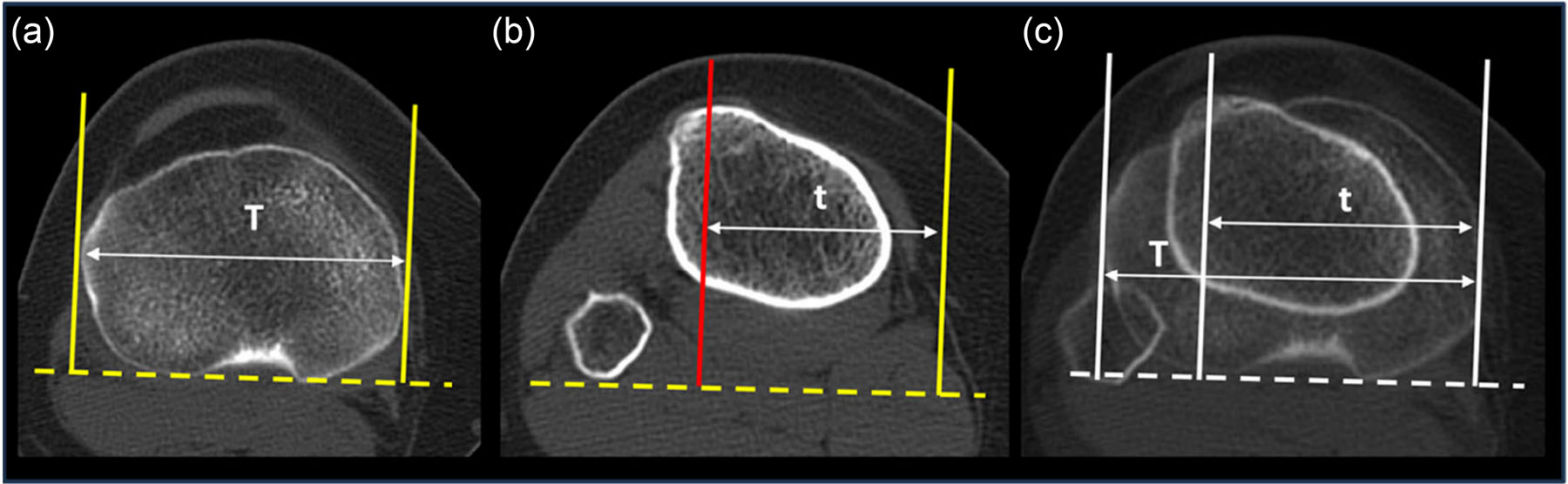
TT lateralisation measurement. (a) Axial CT image of the tibial plateau. The width of the tibial plateau (distance *T*) is the distance between the two lines perpendicular to the posterior bicondylar line passing through the medial and lateral border of the tibial plateau. (b) Axial CT image of the TT. The distance between the centre of the TT and the medial border of the proximal tibial condyle (distance *t*) was measured. TT lateralisation was calculated as *t* ÷ *T*. (c). Addition CT image. CT, computed tomography; TT, tibial tubercle.

**Figure 4:**
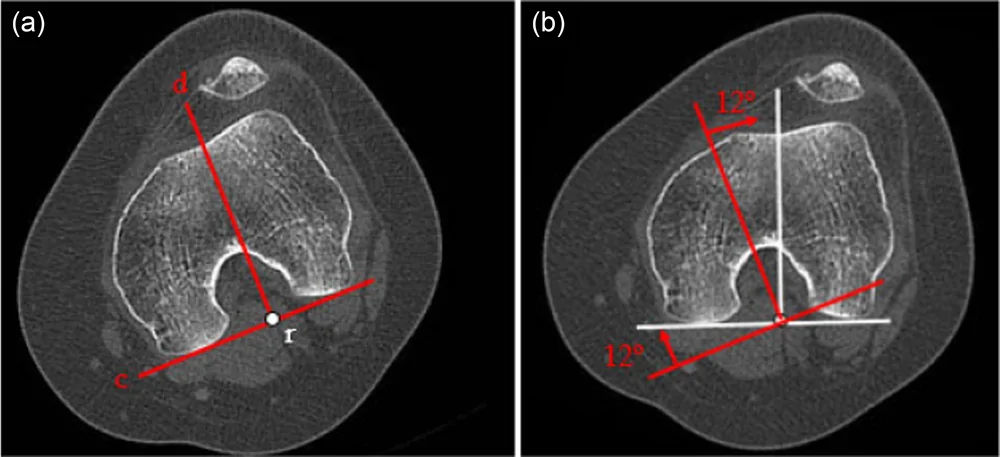
(a) Intersection of the posterior femoral condylar line (c) and the perpendicular to the trochlear groove (d) as the reference rotation point (*r*). (b) Shows the correction to neutral knee rotation angle after applying a correction of 12°.

The following anatomical values were determined: (1) TT‐TG distance: measured between the centre of the trochlear groove and the centre of the TT in the axial plane, according to the technique described by Goutalier et al. [8], (2) KRA: determined using the Takai method [19], calculating the angle formed between the posterior femoral condylar line and the tibial bicondylar line (Figure 1), (3) TT‐PCL distance: measured according to Seitlinger's method [14] in the axial plane as the distance parallel to the dorsal condylar line of the tibia that passes between the medial edge of the posterior cruciate ligament at its tibial insertion and the centre of the patellar tendon insertion on the TT (Figure 2) and (4) TTL relative to the maximum transverse diameter of the tibial plateau and expressed as a ratio of this transverse diameter using the method by Tensho et al. [20] (Figure 3).

**Figure 5:**
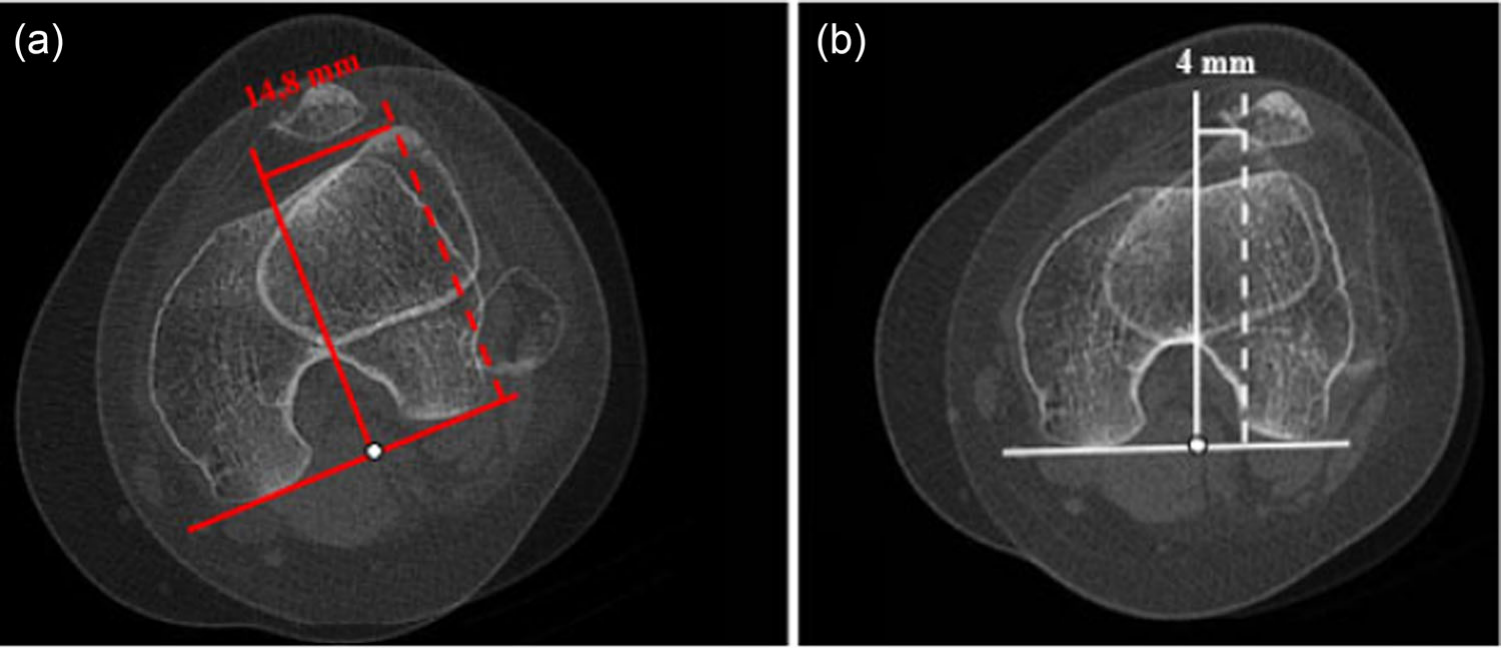
TT‐TG distance according to the technique described by Goutallier [8]. (a) Shows the normal TT‐TG distance. (b) Shows the TT‐TG corrected by the KRA. KRA, knee rotation angle, TT‐TG, tibial tuberosity–trochlea groove.

To analyse the influence of KRA on TT‐TG distance, we established the intersection of the posterior femoral condylar line and the perpendicular to the trochlear groove as the reference rotation point (Figure 4a). From this point, knee rotation was corrected to 0°, after which the TT‐TG distance was recalculated (Figure 4b). This new value is defined as the Rotational‐Corrected TT‐TG distance (RC TT‐TG), which represents the theoretical measurement of the TT‐TG distance in the absence of rotation (Figure 5).

### Statistical analysis

Statistical analyses were performed using IBM SPSS Statistics (version 25). A descriptive analysis was performed, including the mean, standard deviation, median, range and 95% confidence interval. Spearman's bivariate correlations were calculated between KRA and the difference in TT‐TG (ΔTTTG) after correction. Simple linear regression analysis was performed to evaluate the influence of KRA on variation in TT‐TG. The results were expressed using the coefficient of correlation (*R*²) and the regression coefficient (*B*). Interobserver reproducibility was assessed using the intraclass correlation coefficient (ICC) with two factors and total concordance. A statistical significance level of *p* < 0.05 was established. Moreover, RC TT‐TG distance was correlated with rotation‐independent tibial‐based measurements (TT‐PCL distance and TTL ratio).

## RESULTS

**Figure 6:**
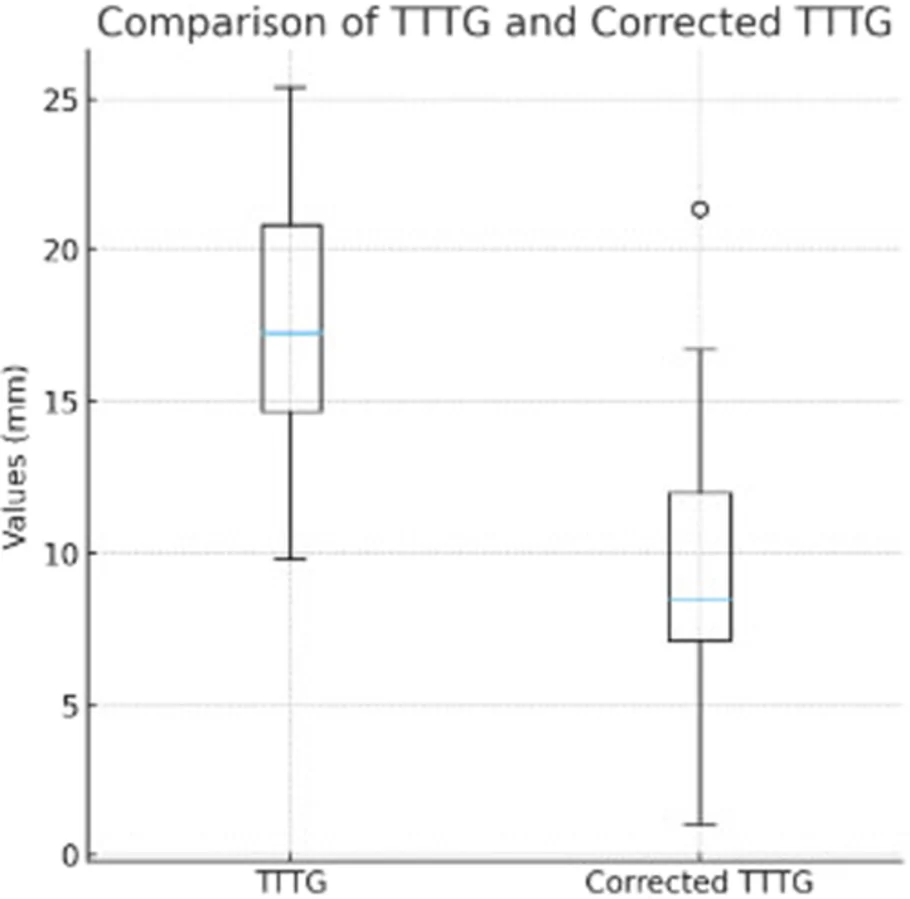
Comparison between normal and corrected tibial tuberosity–trochlear groove (TT‐TG) distances. Boxplot illustrating the distribution of TT‐TG distances before and after correction for knee rotation. The rotational corrected TT‐TG (RC TT‐TG) values are notably lower and show reduced variability compared to the normal TT‐TG, indicating the influence of rotational alignment on measurement accuracy.

Details on patient demographics are reported in Table 1. The measured parameters are shown in Table 2. Normality tests were performed to evaluate the distribution of the variables (Kolmogorov–Smirnov). TT‐TG, KRA did not follow a normal distribution (*p* < 0.05). RC TT‐TG and ΔTTTG followed a normal distribution. The TT‐TG distance had a mean of 17.22 mm, and the corrected TT‐TG distance had a mean of 9.34 mm; the Wilcoxon test confirmed that both measurements differ significantly (*p* < 0.001), indicating that the correction clearly modifies the quantification of the TT‐TG (Figure 6).

**Table 1:** Demographic and clinical characteristics of the patient cohort.

Patients (*n*)	100 (183 lower limbs)
Age (years)	22.7 + 5.9 (18–47)
Limb R/L	92/91
BMI (kg/m^2^)	20.5 ± 2.4 (18.2–33.8)

**Table 2:** Descriptive statistics for tibial tubercle‐trochlear groove (TT‐TG) distance, knee rotation angle (KRA), rotational corrected tibial tubercle‐trochlear groove distance (RC TT‐TG) and difference between TT‐TG distance, rotational corrected TT‐TG (ΔTTTG), tibial tubercle‐posterior cruciate ligament distance (TT‐PCL) and Tibial tuberosity lateralisation (TTL).

Measures (units)	Mean (CI 95%)	Median	Standard deviation	Min–Max	Range
TT‐TG (mm)	17.22 (16.61–17.83)	17	4.20	9.30–29.50	20.20
KRA (grades)	8.81 (8.07–9.55)	8.0	5.08	0–25	25.00
RC TT‐TG (mm)	9.34 (8.66–10.03)	9.4	4.70	0–27	27.00
ΔTTTG (mm)	7.87 (7.14–8.60)	7.9	5.01	−8 to 22.37	30.37
TT‐PCL (mm)	20.32	20	3.35	13–29	16
TTL (%)	0.66	0.66	0.039	0.56–0.77	0.21

In the total sample (*n* = 183), 27.3% of patients had a TT‐TG > 20 mm, while after applying the correction by the KRA, only 2.2% remained above this threshold. In the subgroup with KRA above the mean in our population (≥8.8°), 42.2% showed an uncorrected TT‐TG > 20 mm and none presented pathological values after correction (Table 3). These differences show a very significant reduction in the proportion of cases potentially eligible for TT medialization.

**Table 3:** Comparison of the proportion of patients with TT‐TG distance > 20 mm before and after correction, in the total sample and in the subgroup with KRA above the mean (KRA ≥ 8.8°).

Patients	TT‐TG > 20 mm (%)	RC TT‐TG > 20 mm (%)	*p*‐value
Total sample (*n* = 183)	50 (27.3)	4 (2.2)	<0.001
Patients with KRA ≥ 8.8° (*n* = 90)	38 (42.2)	0 (0)	<0.001

Abbreviations: KRA, knee rotation angle; mm, millimetres; TT‐TG, tibial tuberosity–trochlear groove.

After correction, a very strong and significant correlation was observed between KRA and ΔTTTG (Spearman *ρ* = 0.934; *p* < 0.001). This indicates that an increase in KRA is associated with a greater decrease in TT‐TG.

**Figure 7:**
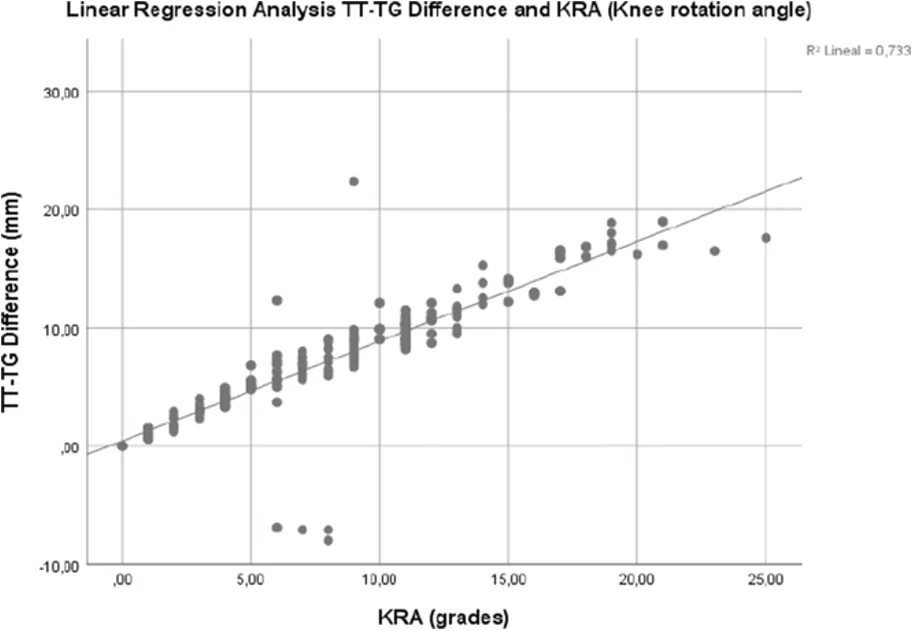
Linear regression analysis between knee rotation angle (KRA) and tibial tubercle‐trochlear groove (TT‐TG) difference. A strong positive linear correlation was observed between the KRA and the TT‐TG difference (*R*² = 0.812). As the KRA increased, the TT‐TG difference also increased, indicating that greater external rotation of the knee is associated with a larger TT‐TG distance. Each dot represents an individual measurement, and the solid line represents the best‐fit linear regression model.

Simple linear regression analysis showed that KRA explains 73,3% of the variability in the ΔTTTG (*R*² = 0.733; *p* < 0.001) (Figure 7). The model indicates that, on average, the TT‐TG distance decreases by approximately 0.84 mm for each degree of knee rotation (*B* = 0.84; *p* < 0.001).

The native TT‐TG distance showed a moderate positive correlation with TT‐PCL (*ρ* = 0.515, *p* < 0.001). In contrast, rotational‐corrected TT‐TG demonstrated a stronger correlation with TT‐PCL (*ρ* = 0.644, *p* < 0.001). The Spearman correlations show a moderate positive association between the normal TT‐TG and TTL (*ρ* = 0.451, *p* < 0.001), while the rotational corrected TT‐TG exhibits a stronger correlation with TTL (*ρ* = 0.539, *p* < 0.001) (Table 4).

**Table 4:** Correlations between tibial tubercle‐trochlear groove (TT‐TG) distance, rotational corrected tibial tubercle‐trochlear groove distance (RC TT‐TG), tibial tubercle‐posterior cruciate ligament distance (TT‐PCL) and Tibial tuberosity lateralisation (TTL).

Correlation	*ρ* (Spearman)	*p*‐value	Interpretation
TT‐TG/TT‐PCL	0.515	<0.001	Moderate
RC TT‐TG/TT‐PCL	0.644	<0.001	Moderate–high
TT‐TG/TTL	0.451	<0.001	Moderate
RC TT‐TG/TTL	0.539	<0.001	Moderate–high

The ICCs between the two observers showed excellent reliability for normal TT‐TG, with an ICC of 0.833 (95% CI: 0.745–0.893). KRA was the most consistent measurement, reaching an ICC of 0.929 (95% CI: 0.888–0.955). The RC TT‐TG showed good reliability, with an ICC of 0.767 (95% CI: 0.650–0.849). All measurements were statistically significant (*p* < 0.001).

## DISCUSSION

The results of this study demonstrate a strong positive correlation between KRA and the variation in TT‐TG distance after rotational correction (*r* = 0.856, *p* < 0.001), with KRA explaining 73.3% of the observed variability (*R*² = 0.733). These results support the idea that TT‐TG distance, as measured in static images, can be significantly affected by rotation between the tibia and femur and that correction for this factor provides a more accurate measurement of TT‐TG distance. If the tibia is externally rotated on the femur, the TT moves laterally and the TT‐TG is increased. This increase in TT‐TG is not a sign of a lateral TT. Therefore, when interpreting TT‐TG values, knee rotation should be taken into account as a potential confounding factor.

Our results are supported by various anatomical and radiological studies [13, 16]. Pascual‐Leone et al. demonstrated in a cadaver model that the TT‐TG distance varies by 0.52 mm for each degree of internal or external rotation of the knee [13]. Similarly, Smith et al. verified an average increase in TT‐TG distance of 0.55 mm (range: 0.5–0.62 mm) for each degree of external rotation of the tibia in computational models [16]. The results of our study are consistent with these findings but provide an in vivo quantification of the magnitude of this effect within a controlled imaging protocol. Our study shows that for each degree of knee rotation, the TT‐TG distance changes by 0.84 mm. Furthermore, the magnitude of the effect observed in our study is greater, probably due to the homogeneity of the sample and the systematic control of position during image acquisition.

Our findings are coincident with some studies that highlight knee rotation as an independent and one of the most significant factors influencing the measurement of TT‐TG distance. Ackermann et al. demonstrated that the KRA is an independent predictor that correlates inversely with ΔTTTG [1]. This finding suggests that when evaluating this measurement, not only flexion but also rotation should be considered. Similarly, Xu et al. identified knee rotation as a major anatomical component associated with increased TT‐TG distance, highlighting its role in the evaluation of patellar instability [23, 24]. Barahona et al. identified a positive correlation between TT‐TG distance and knee articular torsion [2]. This indicates that an increase in external tibial torsion or internal femoral torsion increases TT‐TG distance. Tensho et al. demonstrated that patients with patellofemoral instability had similar values for groove medialization and tubercle lateralisation compared to healthy controls [20]. However, they found greater TT‐TG distances and knee rotations in their patients [20]. Therefore, current evidence suggests that knee rotation and torsional variations are critical factors that must be considered when interpreting TT‐TG distance. Recent studies have found that the difference in TT‐TG values in patients with patellar instability is influenced more by the rotational component (5.3 vs. 1.0 mm) than by the translational component (13.4 vs. 11.0 mm). Furthermore, this difference is present in 73% of cases with elevated TT‐TG, either in isolation (42%) or in combination (31%), compared to 8% with an exclusively translational increase [15]. Other authors have proposed different indices to achieve a more comprehensive and patient‐specific assessment [3, 5, 11]. In this context, the strong association observed between KRA and RC TT‐TG supports the inclusion of axial rotation in TT‐TG interpretation, although the clinical implications of using corrected values remain to be determined. In this regard, RC TT‐TG could be considered an intermediate step toward a three‐dimensional and individualised assessment of patients with patellofemoral disease. Taken together, these findings underscore the importance of considering knee rotation as an essential parameter for accurately interpreting TT‐TG distance [23].

Although several measurements have been proposed to reduce the inherent bias of the TT‐TG distance—such as TT‐PCL and TTL—we aim to refine it by providing a method that allows its interpretation while accounting for the bias introduced by femorotibial rotation. In this context, TT‐TG demonstrated only moderate correlations with tibial‐based measurements, whereas RC TT‐TG consistently showed moderately high correlations with those same parameters. This shift from moderate to moderately high association suggests that correcting for rotation improves the anatomical relevance of the TT‐TG measurement. Overall, these findings support the concept that rotational correction yields a more anatomically meaningful assessment of TTL and highlight the potential value of RC TT‐TG for clinical interpretation and future research.

Our paper could lead to relevant clinical implications in future research. The use of the traditional 20 mm TT‐TG threshold as an indication for TT osteotomy may, in some cases, overestimate the need for surgery in patients whose TT‐TG values fall within the normal range after accounting for limb rotation. This phenomenon could explain why some patients with pathological TT‐TG values initially present normal dynamic alignment on dynamic MRI studies or suboptimal results after TT osteotomy [4, 25]. Correcting TT‐TG based on knee rotation had a significant clinical impact on our population. It reduced pathological values (>20 mm) from 27.3% to 2.2% in the total sample and eliminated them completely in the subgroup with KRA ≥ 8.8°, which corresponds to the mean knee rotation observed. These results suggest that excessive rotation can lead to a significant overestimation of TT‐TG. Several studies have reported mean KRAs of around 8°–9° in both healthy populations and patients with patellofemoral instability [9, 10]. These findings support using this threshold to define the subgroup with high rotation. From a clinical perspective, these results suggest applying TT‐TG correction, especially to patients with KRA above the population average. In patients with lower values, correction may be unnecessary, thus optimising diagnostic accuracy and avoiding overtreatment. One of the main strengths of this study is that, to our knowledge, it is the first to propose direct correction of TT‐TG distance based on knee rotation. This approach may help refine measurement interpretation, but its role in guiding treatment decisions must be validated in prospective clinical studies. A rotational corrected TT‐TG distance value could be a more accurate, more reproducible parameter and potentially more useful for the radiological evaluation of the patient. Our findings are consistent with previous publications and open the door to future studies exploring whether RC TT‐TG is a better predictor of true TT lateralisation or if it can be incorporated into multifactorial risk models.

### Limitations

First, the analysis was performed exclusively on tomographic slices in extension, without considering the impact of the flexion angle on the measurements. Other researchers have shown that this factor can modify the measurement of the TT‐TG distance [10]. Furthermore, although our sample was homogeneous, AKP female patients, and the analysis was controlled in terms of technique and position, the model did not include other anatomical and biomechanical factors that influence the TT‐TG distance, such as femoral anteversion, tibial torsion and trochlear dysplasia. Future studies should integrate these parameters simultaneously using multivariate models to more accurately define the relative contribution of each component. Second, the rotational correction uses two‐dimensional axial CT images, which is a simplification of true knee anatomy. Nevertheless, it offers a practical, reproducible, and clinically feasible method to account for rotational effects, addressing a key source of TT‐TG variability and improving the interpretability of the measurement. Finally, our results are only applicable to AKP female patients and not to other population groups.

## CONCLUSION

Knee rotation is an important factor influencing TT‐TG measurements, with TT‐TG decreasing by approximately 0.84 mm for each additional degree of rotation. Rotational corrected TT‐TG provides a more consistent and anatomically meaningful measurement, which may help improve the interpretation of TT‐TG in individual patients. However, the clinical implications of using rotationally corrected TT‐TG, including potential effects on surgical decision‐making or threshold values, remain to be established and should be evaluated in future clinical studies. Relying on TT‐TG without considering knee rotation may lead to misinterpretation and should be approached with caution.

## AUTHOR CONTRIBUTIONS

**Vicente Sanchis‐Alfonso**: Project coordinator; designing the study; writing the manuscript. **Jesús Castellano‐Curado**: Writing the manuscript; evaluating the radiographs and collecting data. **Marco Gulmini**: Evaluating the radiographs; collecting data. **Cristina Ramirez‐Fuentes**: Evaluating the radiographs; writing the manuscript. **Robert A. Teitge**: Designing the study. **Julio Doménech‐Fernández**: Writing the manuscript and analysing the data.

## CONFLICT OF INTEREST STATEMENT

The authors declare no conflicts of interest.

## ETHICS STATEMENT

The study was approved by the clinical research ethics committee at our institution (CEIm Hospital Arnau de Vilanova, Valencia, Spain # PI 21_2024).

## Data Availability

The data that support the findings of this study are available from the corresponding author upon reasonable request.
